# A methodology for the design of an effective air quality monitoring network in port areas

**DOI:** 10.1038/s41598-019-57244-7

**Published:** 2020-01-15

**Authors:** Luigia Mocerino, Fabio Murena, Franco Quaranta, Domenico Toscano

**Affiliations:** 1 DII – Department of Industrial Engineering, University of Naples “Federico II”, Naples, Italy; 2 DICMAPI – Department of Chemical, Materials and Industrial Production Engineering, University of Naples “Federico II”, Naples, Italy

**Keywords:** Environmental impact, Mechanical engineering

## Abstract

The assessment of the impact of ship emissions is generally realised by a network of receptors at ground level inside the port area or in the nearby urban canopy. Another possibility is the use of dispersion models capable of providing maps of concentrations to the ground taking into account ship emissions and weather conditions. In this work traffic data of passengers ships in the port of Naples were used to estimate pollutant emissions starting from EMEP/EEA (European Environment Agency/European Monitoring and Evaluation Programme) methodology and real data of power engines. In this way, a hourly file of emission rates was produced and input to CALPUFF together with meteorological data. Then SO_2_ concentrations at different heights (0–60 m) in correspondence of selected points within the port area were evaluated. Results are compared with data measured at ground level in monitoring campaigns showing how is possible to better identify and quantify the air pollution from ships in port by positioning the receptors inside the port area at different heights from ground-level. The results obtained give useful information for designing an optimum on-site air quality monitoring network able to quantify the emissions of pollutants due to naval traffic and to individuate the contribution of single ships or ships’ categories.

## Introduction

The effects of pollution connected to ship traffic have impact on a global scale, with the production of greenhouse gases, and on a local scale with the introduction of nitrogen oxides (NO_x_), sulfur oxides (SO_x_) and particulate matter into the atmosphere. The overall production of pollutants in the lower parts of the atmosphere contributes negatively to the quality of the air we breathe with effects on man and environment. Mainly, SO_x_ are emitted as a function of the content of sulphur in the fuel; the quantity of NO_x_ in the exhausts depends on the inner parameters of engines and on the temperature of combustion, timing. In addition, diesel engines emit particulate matter (PM) mostly during transient states of engines in port and these substances are particularly dangerous since they can create the conditions for oncological diseases.

Ships of any type are “producers” of noxious emissions; in fact, the most part of ships has diesel engines to provide the power needed for the propulsion and for the auxiliary services onboard. However, in all cases, engines must be considered a source of pollution since their operations will cause emissions of noxious elements for sure. The “*International Convention on the Prevention of Pollution from Ships*”, MARPOL (*MARine POLlution*) 73/78, is the IMO (*International Maritime Organization*) regulation concerning ship-related pollution^1^.The Annex VI (1997), of the MARPOL, sets limits on NO_x_ and SO_x_ emissions from ship exhausts and prohibits deliberate emissions of ozone from ships of 400 gross tonnage and above. The original annex of MARPOL has undergone successive modifications aimed at reducing the limits related to the emissions of main pollutants in light of the technological improvements made over the years and the ever more stringent need to reduce emissions^2,3^. The main amendment to the regulation saws a progressive reduction in emissions of nitrogen oxide and sulfur and the introduction of controlled emission zones (ECAs) with even more stringent limits^3^. For the NOx emissions, different limits (TIER) have been introduced depending on the year of construction of the ship, the rpm of the engine and the areas where the ship works^3^. To date, the level of reference is TIER II; the TIER III is to be considered valid only in the ECA (*Emission Control Area*) areas^2^. Since the sulphur generating the oxides is present in the fuel (and not in the air as the nitrogen), the limits to the emissions of sulphur oxides concern the mass percentages of sulphur present in the bunkered fuel. From 1 January 2020, the global sulphur cap will be reduced from the actual 3.5% to 0.5% while in the ECA areas; today some zones have a limit of 0.10% (from 1/1/2015) (IMO, 2007).

Many studies concern the assessment of the contribution of ship emissions on air quality in port and nearby urban areas; in several cases a limited contribution of ship emissions inside the port areas is reported. In fact, a large contribution of cruise ship emissions in Victoria’s port (Canada) is observed at about 1 km from the mooring points^4^ and up to 5 km inland at Taranto (Italy)^5^. The highest impact of ship emissions of PM_2.5_ is estimated at 10 km from the coastline in Bohain Rim region (China)^6^ and at about 1 km from the port source in Los Angeles^7^. It is also generally highlighted that the influence of ship emissions on pollutant concentration at ground is statistically relevant only during ship-plume-influenced periods (i.e.; when ship activities occur in correspondence of wind blowing from the port area to the receptor site). This finding comes from measurements of O_3_, NOx, SO_2_ and PM_2.5_ in Brindisi^8^, of PM_2.5_ in Shangai^9^, in Bohain Rim region^6^ and in Los Angeles^7^ and, finally, of SO_2_ in Victoria (Canada)^4^. Results indicate that ships can contribute to 20–30% of the total PM_2.5_ but only during ship-plume-influenced periods^9^, and about 11% at 10 km from the coastline. The strong time and wind dependence of the impact of ship emissions at ground level is the reason why some correlation studies use short averaging time measures (from 1 to 10 minutes)^10,11^. On the contrary, other studies estimate a significant contribution/impact inside the port area. Yau *et al*.^12^ in Hong Kong using the PMF (*Positive Matrix Factorization*) source apportionment method observe that at the container terminal the PM_2.5_ contribution of vessels to period average mass concentration can be up to 25%. Kontos *et al*.^13^ found that the maximum concentration of NO_2_ is located near the passenger terminal and assumes the value of 270 μg/m^3^ representing 135% of the hourly average limit value (LV) value (200 μg/m^3^). Sorte *et al*.^14^, using the C-PORT model, assessed that the highest concentrations of PM_10_ were found inside the Leixões port area. The different findings corresponding to the localization of the area of maximum impact of ship emissions are not surprising, in fact it depends on several factors: extension of the port area, the distance of the urban area, the height of funnels, wind conditions. To better characterize the effect on a local scale of the ship’s emissions in port, dispersion models can be very useful. In the present work we adopted the software CALPUFF^15^, as dispersion model. It has been often used in studies of the impact of ship emissions: (i) Poplawski *et al*.^4^ investigated the impact of cruise ships on level concentrations of PM_2.5_, NO_2_, SO_2_ in James Bay, Canada. (ii) Cruise and Passenger Ship Air Quality Impact Mitigation Actions^16^ project assessed the impact on air quality and the risk to the health of the population associated with the maritime traffic of passenger and cruise. The analysis is performed for present and future scenarios with and without mitigation actions that are examined in the five port: Barcelona, Genova, Marseilles, Venice and Thessaloniki^13,16^ (iii) Murena *et al*. ^17^ evaluated the impact of cruise ships in 2016 on the urban area of Naples. The results obtained in this study of the assessment of the impact of cruise ship emissions^17^ show a significant contribution at peak concentrations episodes: the contribution to the 99^th^ percentile of 1-hour NO_2_ concentrations can reach 86.2% during high season (June-September). The relatively low levels of pollutants, observed or simulated, in the port of Naples, may be due to the short distance of the monitoring points in port in relation to the mooring points.

As a matter of fact, the effective emission height (height of funnels plus the plume rise) for cruise ships and largest ferries may reach 60–70 meters. As a consequence, their emissions impact the ground level at a distance of about hundred meters from the releasing point. Monitoring the concentration at higher heights could be an alternative to ground level. Information on vertical profiles of pollutant concentrations in the port of Naples are not available. Only vertical average values of PM_2.5_ are reported in a monitoring campaign using drones^18^. Results show how vertical average values are strongly dependent on the horizontal coordinates, namely the position inside the port area. Anyway, the port area is doubtless the best choice to locate receptor points of a monitoring network to assess and control ship emissions. In fact, this choice minimizes the need for authorization and the overlap of all the typical gas pollutants sources normally present in an urban area: traffic, heating, domestic, commercial emissions and others. However, these results show that, if you want a better characterization and the apportionment of the sources of pollution isolating the primary pollution due to port traffic activities, the best position of receptors is not always at ground level inside the port area.

Data collected in two periods of about 15 days each are reported and analysed in this paper: one (March-April 2012) characterized by low traffic and the other (November 2012) by high traffic of cruise ships.

## The Case Study: the Port of Naples

**Figure 1:**
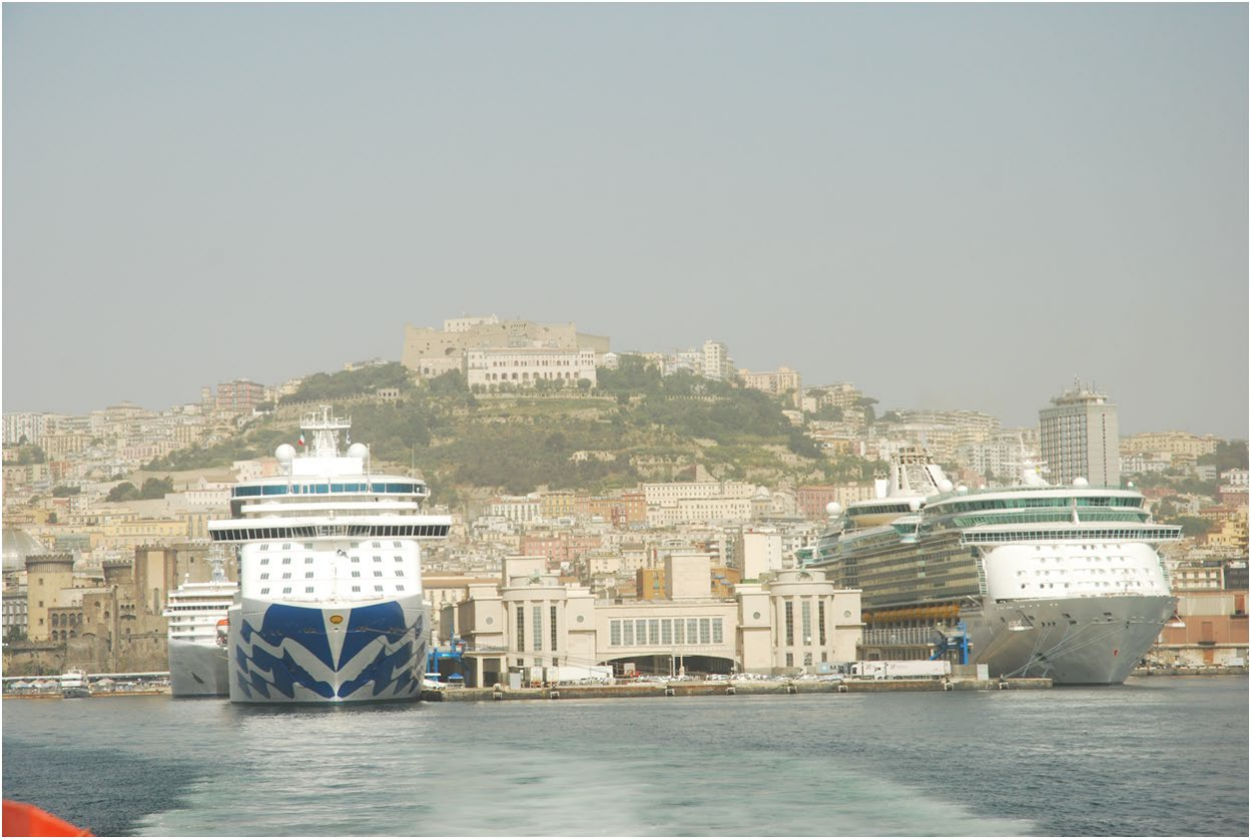
The port of Naples.

The port of Naples, Fig. 1, is embodied in the Authority of Harbour System of the Central Tyrrhenian Sea, governing body of the Campania’s port system, which includes the harbors of Naples, Salerno and Castellammare di Stabia. The port of Naples has always been a crossroads for exchanges throughout Europe. The traffic of goods and passengers affecting the port of Naples have seen a strong growth in recent years. According to the data updated to 12/31/18, the number of containers increased by 10% compared to 2017. The Neapolitan terminal is busy firstly by the traffic of passengers from ships connecting with the small islands and those with Sicily and Sardinia; great importance (and big dimension) has the passenger movement due to the cruise ships. Data updated to December 2018 show a +15.23% of cruise passengers compared to 2017 with a peak of +16.66% in the first half of 2018 (from 927.458 in 2017 to 1.068.797). The Cruise Terminal has seven piers 1,100 meters long and 11 meters of maximum depth with seven movable gangways^19^. Numerous cruises stop every year, especially in the periods of June-July and September-October when it is not impossible to have up to five ships simultaneously present in the port. Several researches have been recently developed in this port: a study assessed the acoustic impact of a Ro/Ro (*Roll on-Roll off*) pax ferry, in manoeuvre and at bollard, in surrounding areas inside the port^20^; Murena^17^ analysed the fallout of pollutants emitted by cruise ships during 2016; Langella *et al*.^21^ analysed the effect of the changeover fuel on global emissions.

## The Period Selected for Simulation

**Figure 2:**
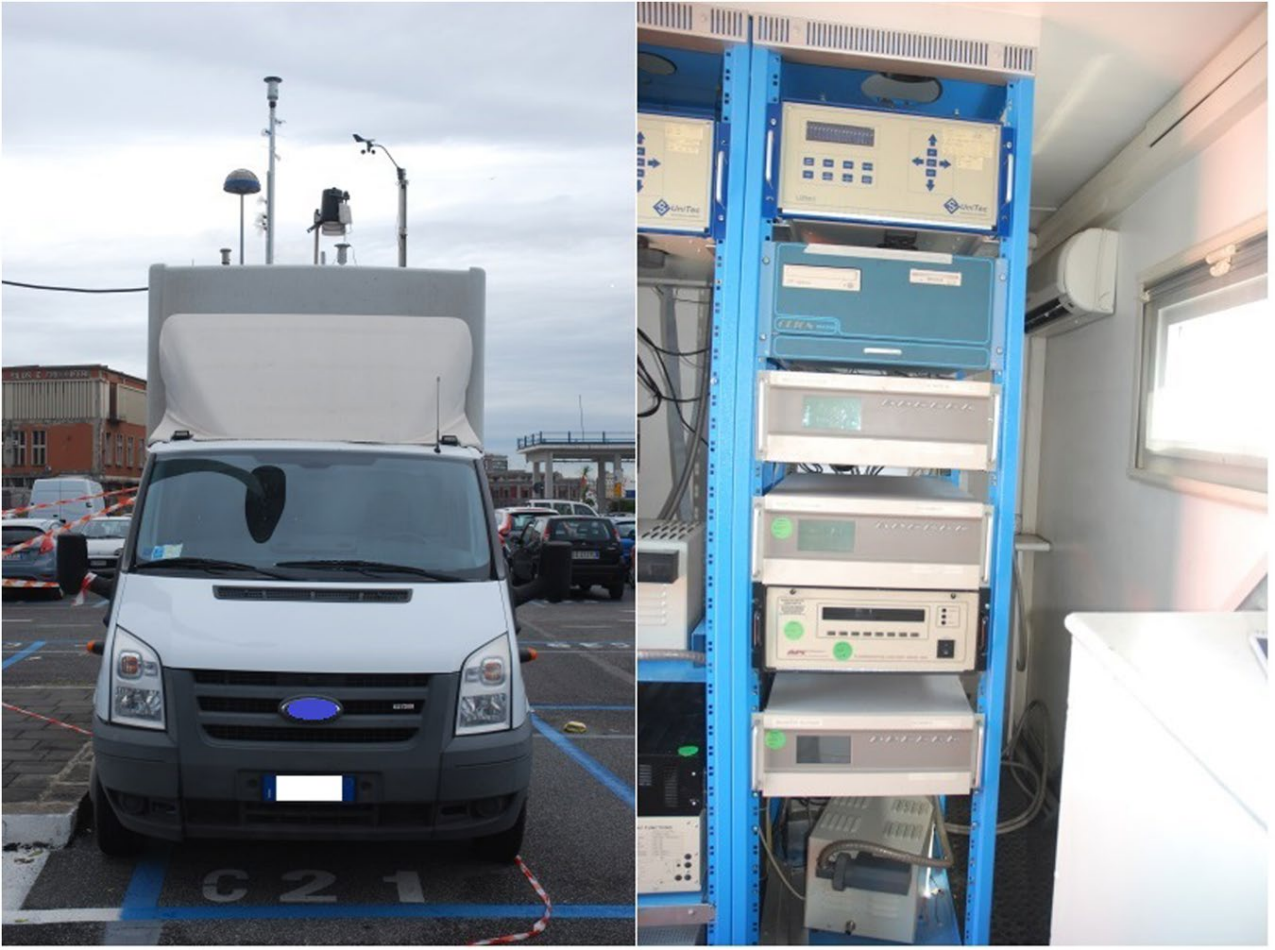
Receptors.

To present the methodology object of this paper, we make reference to data of monitoring collected during 2012^22^. Two periods of about 15 days were studied corresponding to two periods of the year when the presence of cruise ships was relatively low (March-April) and high (October-November). So, the first campaign has been performed in the period between March 28^th^ – April 10^th^; the second between the 2^nd^ and 14^th^ of November. In both cases, ships were located mostly between the wharves n.5 and 11, some in 21 and 22; the instrumented van together with instruments are represented in Fig. 2. Data about the instruments and the results of the monitoring campaigns are detailed in^22^.

For all pollutants considered, the hourly concentration averages have been obtained in both monitoring campaigns of 2012^22^. The results are synthetically reported in Table 1. If compared with limit values established by European directives, they cannot be exhaustive due to the limited time of monitoring campaigns. However, it can be observed that: short time averaged values (1 hr, 8 hours and 24 hours) do not never exceed the limit values established; period averages measured may be compared with year average limit values (in this case, the value of concentration of NO_2_ in March-April campaign raised up to 48.4 μg/m^3^ so exceeding the limit of 40 μg/m^3^). The results obtained by campaign in 2012 showed that the mean daily concentration of NO_2_ and PM_10_ were always lower than the respective LVs (*Limit Values*). Period average concentrations of PM_10_ and benzene were lower than the respective annual average LVs. On the contrary, period average concentration of NO_2_ (41.1 μg/m^3^) slightly exceeded the annual limit value (40 μg/m^3^). SO_2_ concentrations were much lower than the hourly and daily average LVs. Moreover, concentration levels of NO_2_ and PM_10_ were comparable to those recorded in the urban area of Naples in the same period.

**Table 1:** Results of the monitoring campaigns.

Pollutant	Averaging Time	Statistical Parameter	Monitoring campaign	
			March - April	November
NO_2_ (μg/m^3^)	1 h	Maximum	156.6	84.2
	Period	Average	48.4	35.8
SO_2_ (μg/m^3^)	1 h	Maximum	26.6	35.5
	24 h	Maximum	3.7	2.5
PM_10_ (μg/m^3^)	24 h	Maximum	40.8	44.2
	Period	Average	27.2	31.6
CO (mg/m^3^)	8 h mobile	Maximum	0.1	0.9

## A Simulation Study for the Optimization of Receptor Sites Inside the Port Area

**Figure 3:**
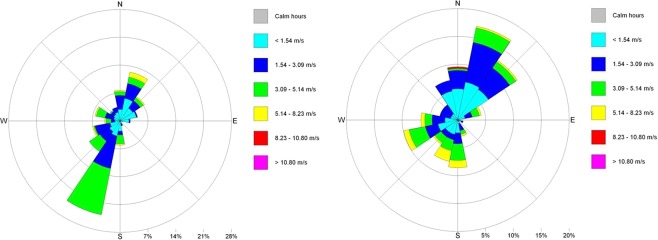
Rose Meteorological data: Left, March-April; right November.

The meteorological characterization of the area is fundamental for the simulation of the transport of pollutants emitted into the atmosphere. 3D wind field data have been obtained starting from the orography of the area with LANDUSE® software and WRF model (Weather Research and Forecasting)^23^. In this way, 3D hourly average values of meteorological parameters were obtained. Wind rose graphs in the monitoring days are reported in Fig. 3. As can be seen, the prevailing direction in March-April are from SSW, while in November the prevailing direction is from NE (Nord-Est).

**Figure 4:**
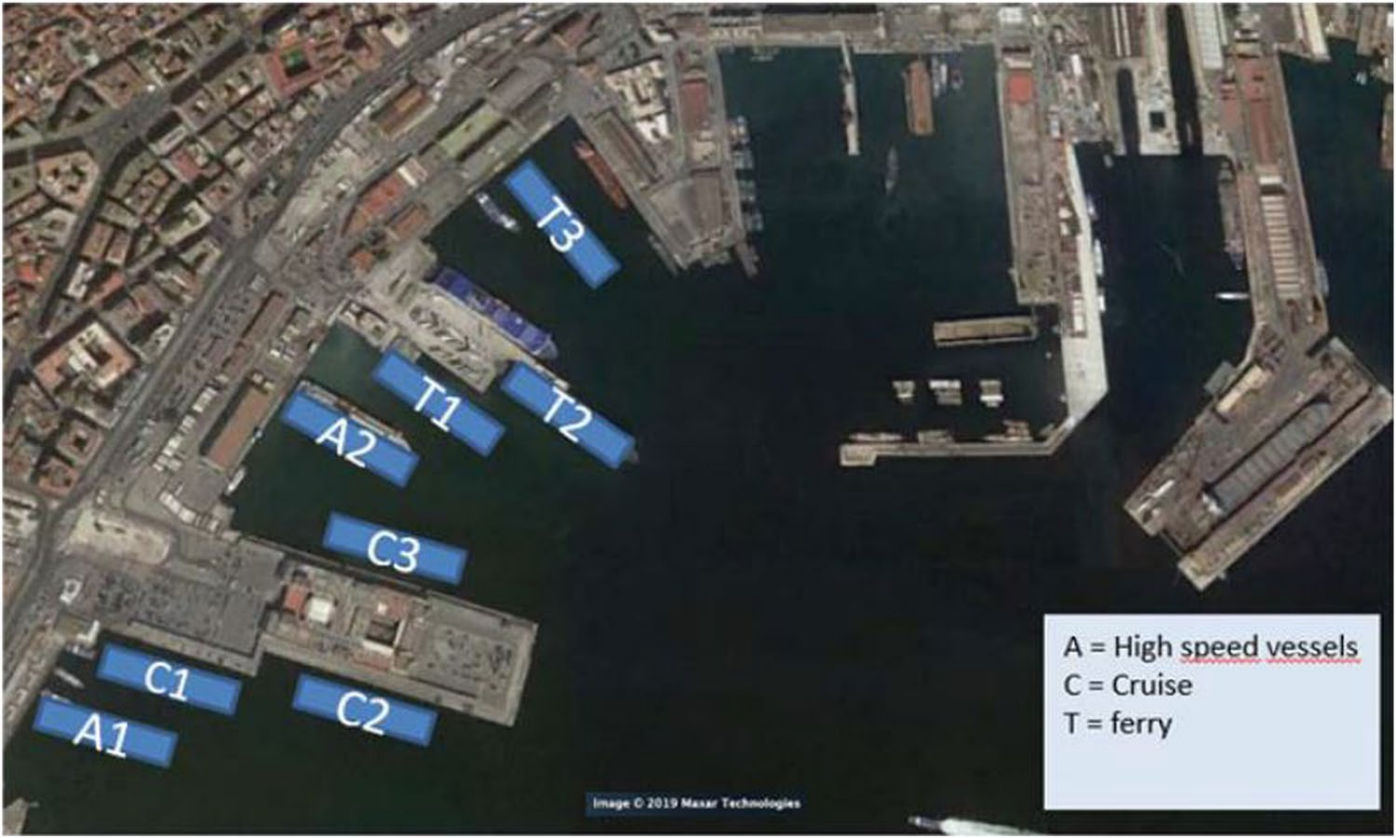
Mooring zones (Images ©2019 Google, Images ©2019 CNES/Airbus, Maxar Technologies, Cartographic Data ©2019).

The geographic coordinates of mooring points were taken from the port map of Google Satellite-based. At each ship the most frequent mooring point was assigned. For small ships (hydrofoils) the very close mooring points have been simplified in a single mooring point being negligible the effect of this different position Fig. 4 dedicated to cruise, high speed vessel and ferry.

**Figure 5:**
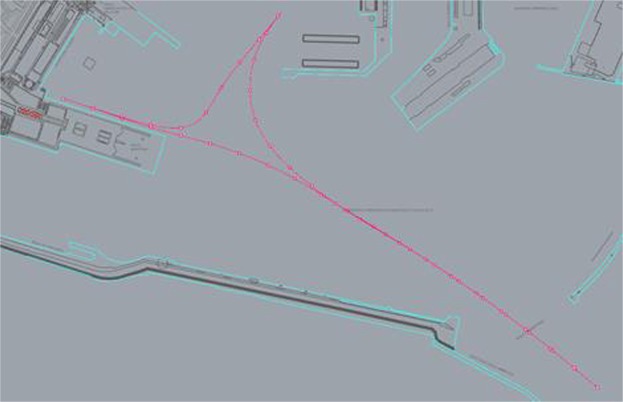
Route for cruise in C3.

For each mooring point, distinct routes have been assumed on which we have then positioned the chimneys as sources of emissions in the phases of maneuvering and transit in port. For all the vessels present in the port, a transit phase was assumed, both in arrivals that in departures, with speeds never exceeding 3 kn. This speed has been set at a reasonable value considering the times employed by a cruise ships from the port mouths to the mooring point Fig. 5.

The manoeuver phase has been inserted always in the entry route of each ships. Therefore, we have assumed that the outbound route performed at a constant speed and almost straight. For the cruise ships the duration of the maneuver has been fixed at 20 min while for the ferries and fast vessels at 10 min. The emission of the ships in the various operational phases in port (transit, maneuvering and mooring) is one of the main input parameters of the dispersion model. A first macro-distinction shall be made between cruise ships, ferries, and fast vessels. The first step was the characterization of each type of vessels on the basis of the total power installed on board. For the maneuvering phases, a load of 20% on the auxiliary engines was assumed, and 50% on the main engine and a duration of 10 min operations (with consumption of 217 and 223 g/kWh respectively for auxiliary and main engine^24^. For these small boats, the emissions during the mooring in port have been supposed to be negligible compared to those of the cruise due to the very high demands for electric power of these ships. For the phase of transit in port, a rate of power, that the vessel will reasonably require for a propulsion with a speed of 3 kn, has been sets. Based on the simulations already carried out^17^, for the cruise ships about 13% of the total installed power was considered during the stops in port to satisfy the hoteling services. During the transit phase, the required power was calculated as the sum of the power necessary to maintain a speed of 3 kn and the power needed for the same on-board services present in the mooring phase. For the maneuver phase, instead, the engine load was divided between main and auxiliary, according to the EMEP/EEA prescription^24^: a load on the ME of 20% and on the AEs of the 50% was assumed with consumption of 223 and 217 g/kWh respectively and a total duration of 30 min. Finally, once evaluated the power in the various phases, for all ships the emission rates in g/s of NO_x_ have been estimated by the emission factors of EMEP/EEA. Emission rate of SO_x_ were evaluated assuming the use on board of fuels to 0.1% by mass of sulphur.

Dispersion simulations were performed by using the modeling chain composed by WRF, CALMET, CALPUFF.

The WRF model is built with a single domain. This domain is centred over the Gulf of Naples and consists of 90 columns and 90 rows of 2 × 2 km^2^ grid cells. The vertical structure of the model includes 50 layers (eta levels) covering the whole troposphere. The WRF simulations were conducted with NCEP (*National Centres for Environmental Prediction*) Global Tropospheric Analyses with 1° × 1° spatial resolution and temporal resolution of 6 h.

Several physics options in WRF are available for: Planetary Boundary Layer, Surface Layer, microphysics, Land-surface and radiation. A list for each schemes using for each parameter are reported in Table 2.

**Table 2:** Parameters used in WRF simulations.

Parameter	Option
Planetary Boundary Layer (*PBL*) scheme	Mellor-Yamada-Janjic scheme (MYJ)
Surface Layer scheme	Similarity theory (Eta)
Microphysics	WRF Single-Moment 6-class scheme
Land-surface model	Noah Land Surface Model
Longwave Radiation scheme	Rapid Radiative Transfer Model
Shortvawe Radiation scheme	New Goddard scheme

The data in WRF output files were interpreted and converted to a 3D.DAT file by CALWRF program. This file is used as the initial guess for meteorological field of CALMET.

In CALMET, we used the recommended parameters by Barclay *et al*.^25^ with the parameters reported in Table 3. The orography in the calculation domain was evaluated by using the software LANDUSE®. The orographic file together with a prognostic file obtained with WRF, are supplied as input to CALMET which produces the 3D weather file adjusting the meteorological field considering the local influence of high resolution data of terrain and land use.

**Table 3:** CALMET options using 3D.DAT files.

Parameter	Recommended Value
NOOBS	2
ICLOUD	4
IPROG	14
TERRAD	2.7 km

CALPUFF is a multi-layer, multispecies, non-steady state Lagrangian Gaussian puff dispersion model that can simulate the effects of temporally and spatially variable meteorological conditions from point, line, area and volume sources. In this case meteorological fields for reference year 2016 were generated by CALMET model for a Cartesian grid, centered on the port site and subdivided into a 200 × 200 cells grid system with 50 m cell spacing. The CALMET vertical grid system considers 10 layers up to 3000 m height. To model the input of emissions in the calculation domain, 85 point sources (corresponding to ships funnel) have been defined. Eight in correspondence of mooring points. The remaining 77 point sources were placed along the arrival and departure courses to simulate emissions during maneuvering and navigation in port. Data on funnel height from sea level and diameter for each vessel category are reported in Table 4. For ferries three different funnel’ heights were assumed depending on the gross tonnage.

**Table 4:** Diameter and funnel height from sea level for each vessel category.

Vessel category	Funnel height [m]	Funnel diameter [m]
Hydrofoils	5	0.5
Cruise ships	40	1
Ferries	15-25-40	1

The exit gas velocity was assumed at 10 m/s for all vessel categories. With all these data a file PTEMARB (Point Source Emissions File With Arbitrarily Varying Emissions) with hourly emission rates of each source point was created and given as input to CALPUFF. Chemical transformation module RIVAD/ARM3^25^ was adopted to simulate chemical reactions of NO_x_ and SO_x_ in the atmosphere. Data for ozone required by the model RIVAD/ARM3 were obtained from the air quality monitoring network of Naples, while default values have been assumed for NH_3_, since local data are not available.

## Results

**Figure 6:**
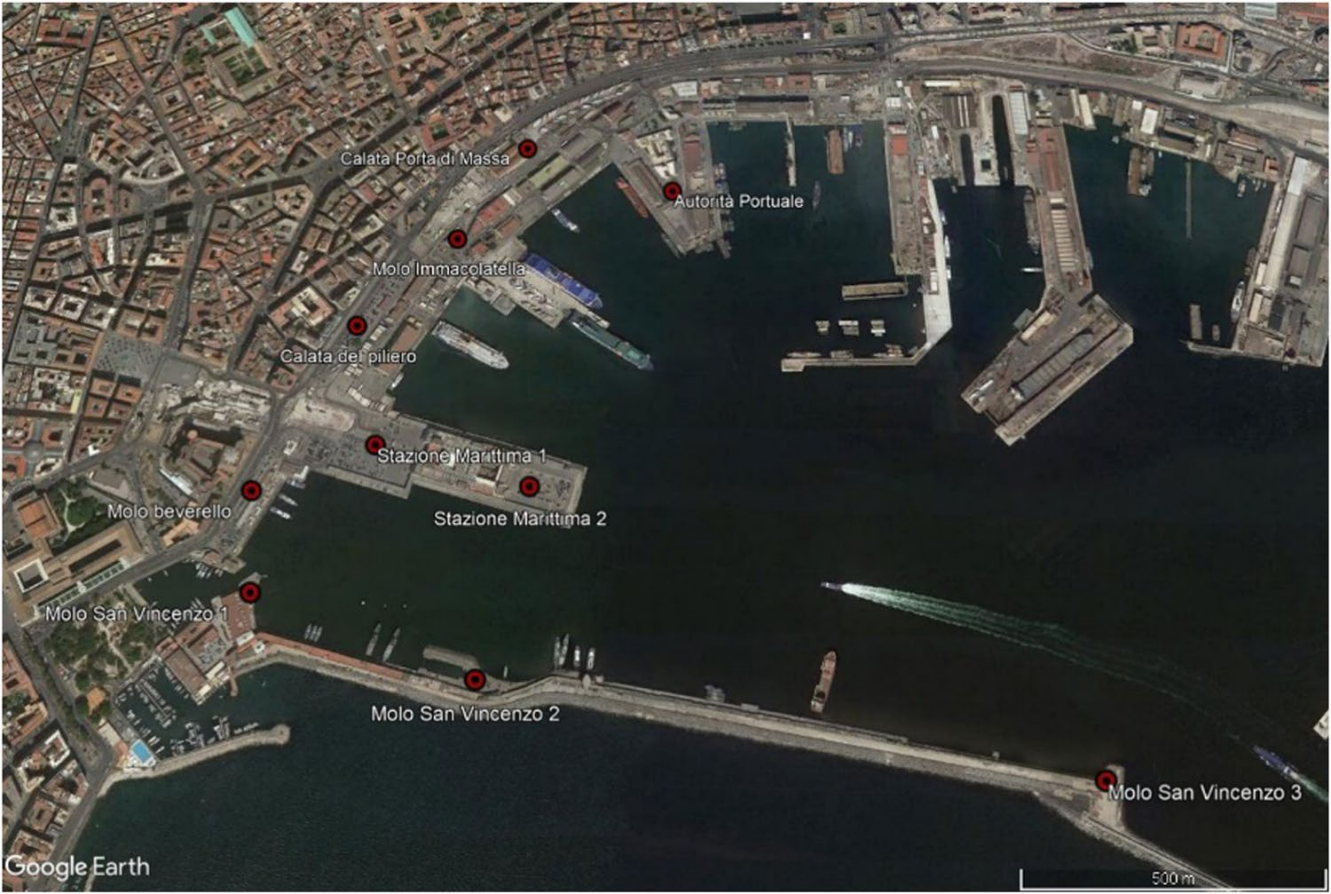
Selected points in the port area for SO_2_ vertical profiles (Google^IT^, Data SIO, NOAA, U.S. Navy, NGA, GEBCO).

**Figure 7:**
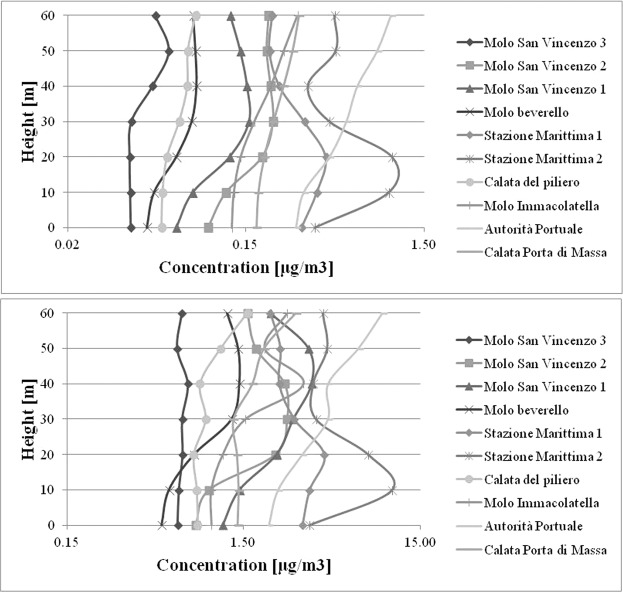
SO_2_ vertical profile concentration, March-April: Up Period Average; Down 98° Percentile.

**Figure 8:**
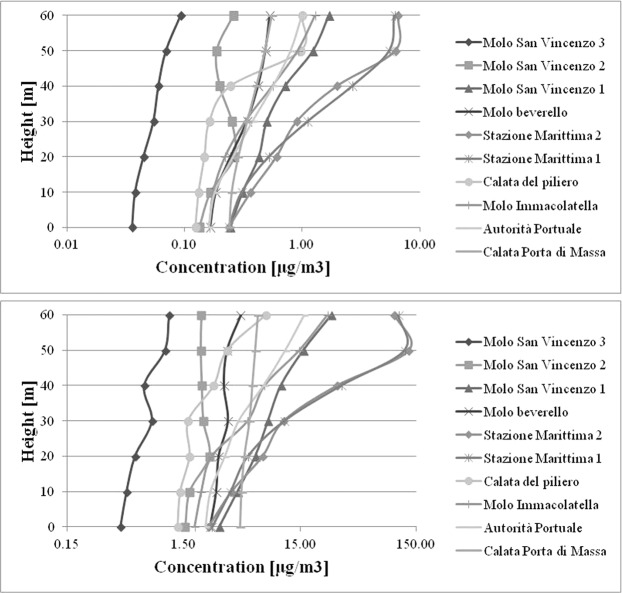
Vertical profiles of SO_2_ concentration, November: Up Period Average; Down 98° Percentile.

Concentrations are calculated by CALPUFF at selected points inside the port area (Fig. 6). To better show how the pollutants emitted by ship funnels are transported in the atmosphere, vertical profiles of SO_2_ are reported in Figs. 7 and 8. Similar results are obtained for NO_2_. The emissions include all the three phases: navigation in port, maneuvering and hoteling and all the passenger ship categories. The results show clearly that for all points selected concentrations at ground level are at a minimum with respect to those at higher height. These finding agrees with previous^22,26^ that indicate a limited impact of ship emissions if measured at ground level inside the port area of Naples. This is a confirmation that ground level is not the best choice as receptors’ position inside the port area. It is also possible to observe that the impact increases with height. However, some differences exist among the different locations. In fact, selected points can be classified into three categories with respect to SO_2_ concentration profile: moderately affected by height; highly affected by height; showing more than a maximum. At the first category belong the points where the impact of ship emissions is limited (e.g. *Calata del Piliero* in March-April). At the second one the points most affected by emissions from elevated funnels of cruise and ferry vessels (e.g. *Stazione Marittima 1* and *Stazione Marittima 2* in November); At the third one the points affected from emissions of both cruise or ferries and fast vessels (e.g. *Molo San Vincenzo 3* in March-April). As can be observed the vertical profiles for the same point in the two periods may be different (e.g. *Molo Immacolatella*). The main differences among all the vertical profiles are the absolute value of SO_2_ concentration and the shape of the vertical profiles.

**Figure 9:**
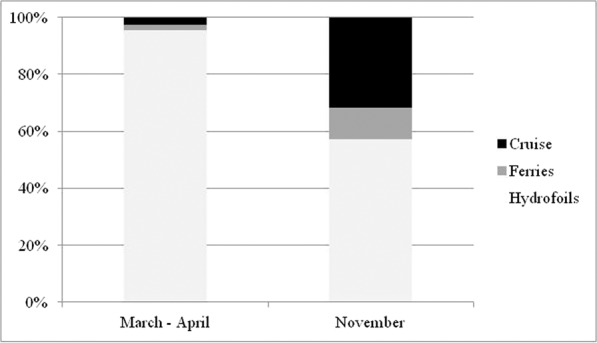
Contribution at SO_2_ airborne concentration of vessel categories at ground level (“Stazione Marittima 1”).

Generally, in March-April the values of concentration are lower than November. In fact, the maximums of vertical profiles are about: 10 μg/m^3^ in March-April and 128 μg/m^3^ in November. For the 98° percentile at the same points the concentrations at ground level are 3.5 μg/m^3^ in March-April and 2.5 μg/m^3^ in November; 1 μg/m^3^ in March-April and 7 μg/m^3^ in November for the period average, instead the concentrations at ground level are 0.4 μg/m^3^ and 6.5 μg/m^3^ respectively in March-April and November. This depends on the different number of calls of cruise ships in the two periods: 19 calls in March-April (14 days) and 26 calls in November (12 days). The differences in the shape of vertical profiles depends on the rose wind pattern in the period. The results of the vertical profiles, for both periods, show that the receptor with the highest concentration level in the period March-April (Fig. 7) is “*Stazione Marittima 2*”, calculated at 20 m, while in November (Fig. 8) the receptors with maximum concentration are: “*Stazione Marittima 1*” and “*Stazione Marittima 2*” in both cases at 50 m. The difference in the height of maximum concentration is probably due to the different contribution of the ship categories in consequence of the rose wind pattern in the period. In fact, the prevailing direction in March-April is from SSW (Sud-Sud West) and the receptor at the “*Stazione Marittima 2*” is downwind respect the emission of hydrofoils (A1 in Fig. 4). In November, instead, the prevailing direction are from NNE (Nord-Nord Est) and NE (Nord Est) and the receptor is downwind respect to the emission of cruise ships at berth C3 and ferries anchored at berths T1 and T2 (Fig. 4). A confirmation of the different contribution of vessel’s categories on vertical profiles is obtained performing specific simulations for each category of vessels: cruise, ferries and fast vessels. Results are reported in Fig. 9 at the receptor point “*Stazione Marittima 1*”. Even though, the emissions of cruise ships are much higher than those of ferries and fast vessels; their contribution at SO_2_ concentration is negligible in March-April and limited in November. The highest contribution being due to fast vessels emissions. It must be highlighted that this source is the most difficult to model. In fact, mooring point and maneuvering route are often variable and the emission height is generally unknown because in many cases a real funnel does not exists.

Our results are based upon a sample size of two periods of about 15 days. However, the shipping traffic emissions could be variable in other periods and the conclusions might be different. Considering the high cost of certified reference instruments, there is a current trend worldwide to increase the spatial and temporal data resolution and range using low-cost air pollutant sensors/monitors^27,28^. Utilizing low-cost air quality platforms in data collecting would be helpful in more adoptive air quality monitoring network design.

## Conclusions

Data of monitoring campaigns collected in several times inside the port area of Naples showed a limited impact of ship emissions on air quality for the main pollutants: SO_2_, NO_2_, PM_10_, Benzene. This evidence is confirmed by simulations with CALPUFF for SO_2_ and NO_2_ emitted by passenger ships. However, this finding does not give information on the actual impact of ship emissions on the urban area of Naples. In fact, due to their height and especially of large cruise ships and ferries, plumes released by ship funnels, can impact at larger distance than that of port area boundaries. In this article, we studied the impact of passenger ship emissions, using the chain model WRF, CALMET and CALPUFF to evaluate the vertical profiles of SO_2_. Two different periods in 2012 were analyzed: 28^th^ March–10^th^ April 2^nd^–14^th^ November. The first period is characterized by a low number of calls of cruise ships while the second by a much higher number. The results clearly show that for all selected points the concentrations at ground level are very low if compared to those at higher height. This is a confirmation of the limited impact of ship emissions inside the port area of Naples at ground level. In fact, the ratio maximum concentration/concentration at ground level ranges are between 1 and 52. In some cases, the highest concentration level is at 20 m, in other cases the maximum calculated concentration is at 50–60 m. This is mainly due to the different height of emissions of vessel categories. Fast vessels due to the small effective height of emission determine a maximum at about 20 m, while cruise ships and large ferries at about 50–60 m. Therefore, where the impact of fast vessels emissions is predominant the maximum concentration is at 20 m, where cruise or large ferries emissions prevail the maximum is at 50–60 m height. As a conclusion, results show that the ground level is not the best choice to allocate receptors within the port area because, depending on the zone, the best choice is between 20–50 m. The methodology proposed can be applied to all ports to obtain very useful information for defining the best position of receptor points of monitoring campaigns or of a monitoring network.
